# 淋巴细胞变异型高嗜酸性粒细胞综合征疗效和生存分析

**DOI:** 10.3760/cma.j.cn121090-20250109-00019

**Published:** 2025-07

**Authors:** 士强 曲, 宁宁 柳, 铁军 秦, 泽锋 徐, 冰 李, 丽娟 潘, 蒙 焦, 清妍 高, 慧君 王, 晓非 艾, 志坚 肖

**Affiliations:** 1 中国医学科学院血液病医院（中国医学科学院血液学研究所），血液与健康全国重点实验室，国家血液系统疾病临床医学研究中心，细胞生态海河实验室，天津 300020 State Key Laboratory of Experimental Hematology, National Clinical Research Center for Blood Diseases, Haihe Laboratory of Cell Ecosystem, Institute of Hematology & Blood Diseases Hospital, Chinese Academy of Medical Sciences & Peking Union Medical College, Tianjin 300020, China; 2 天津医学健康研究院，天津 301600 Tianjin Institutes of Health Science, Tianjin 301600, China

**Keywords:** 淋巴细胞变异型, 高嗜酸性粒细胞综合征, 临床特征, 糖皮质激素, 干扰素, Lymphocytic variant, Hypereosinophilic syndrome, Clinical features, Glucocorticoids, Interferon

## Abstract

**目的:**

分析淋巴细胞变异型高嗜酸性粒细胞综合征（L-HES）患者的临床特征、疗效和生存。

**方法:**

回顾性收集2019年7月至2024年10月期间中国医学科学院血液病医院连续诊断的16例L-HES患者资料，以同期诊断的65例特发性高嗜酸性粒细胞综合征（iHES）患者作为对照组，比较两组患者的临床和实验室特征，以及患者的临床疗效和生存。

**结果:**

L-HES患者就诊时最常见的受累器官分别为皮肤（75.0％）、胃肠道（25.0％）、呼吸道（18.8％）、淋巴结（18.8％）、心脏（12.5％）和脾脏（6.3％）。同iHES组患者相比，L-HES患者皮肤受累比例更高（*P*＝0.016），其他器官受累差异无统计学意义。两组患者全血细胞计数中的各项参数差异无统计学意义。通过多参数流式细胞术检测，L-HES组患者外周血中CD3^-^CD4^+^T细胞占淋巴细胞中位百分比为4.08％（*IQR* 1.64％～32.78％），中位绝对计数为0.10（0.05～0.55）×10^9^/L。L-HES组患者的血清免疫球蛋白（Ig）E水平显著高于iHES组（*P*<0.001）。75.0％的L-HES患者检测到T细胞受体（TCR）基因克隆性重排。诊断后有14例L-HES患者接受糖皮质激素作为一线治疗，总有效率为92.9％。在糖皮质激素减量过程中，11例患者再次出现嗜酸性粒细胞计数升高或临床症状加重，3例患者接受α干扰素作为二线治疗，2例获得完全缓解。中位随访16（*IQR* 8～28）个月，1例患者于就诊后8个月死于心功能不全，未发现淋巴瘤转化的病例。2年总生存率为（91.7±8.0）％，同iHES组的（96.2±2.6）％比较，差异无统计学意义（*P*＝0.746）。

**结论:**

L-HES患者预后良好，就诊时容易表现为皮肤受累和血清IgE显著升高。患者通常对糖皮质激素治疗较敏感，但减量过程中容易出现复发，干扰素是一种有效的二线治疗药物。

## Abstract (en)

**Objective:**

To analyze the clinical characteristics, therapeutic responses, and survival outcomes of patients with lymphocytic variant hypereosinophilic syndrome（L-HES）.

**Methods:**

We retrospectively reviewed clinical data from 16 consecutive patients diagnosed with L-HES at the Institute of Hematology and Blood Diseases Hospital, Chinese Academy of Medical Sciences, between July 2019 and October 2024. A control group of 65 patients with idiopathic hypereosinophilic syndrome（iHES）, diagnosed during the same period, was used for comparison. Clinical and laboratory characteristics, therapeutic responses, and survival outcomes were compared between the two groups.

**Results:**

The most frequently involved organs at presentation in patients with L-HES were the skin（75.0％）, gastrointestinal tract（25.0％）, respiratory tract（18.8％）, lymph nodes（18.8％）, heart（12.5％）, and spleen（6.3％）. Compared with iHES patients, patients with L-HES had a significantly higher incidence of skin involvement（*P*＝0.016）, with no statistically significant differences observed in the involvement of other organs. No statistically significant differences were found in complete blood count parameters between the two groups. Multiparameter flow cytometry revealed that the median percentage of CD3^-^CD4^+^ T cells in the peripheral blood of patients with L-HES was 4.08％（*IQR*: 1.64％–32.78％）, with a median absolute count of 0.10（0.05–0.55）×10^9^/L. Serum immunoglobulin E（IgE）levels were significantly higher in the L-HES group than in the iHES group（*P*<0.001）. Clonal rearrangement of T-cell receptor genes was detected in 75.0％ of patients with L-HES. After diagnosis, 14 patients with L-HES received glucocorticoids as first-line therapy, yielding an overall response rate of 92.9％. During glucocorticoid tapering, 11 patients experienced recurrent eosinophilia or worsening of clinical symptoms. Three patients received interferon-alpha as a second-line therapy, with two achieving complete remission. After a median follow-up of 16 months（*IQR*: 8–28 months）, one patient died of cardiac insufficiency 8 months after diagnosis, and no cases of lymphoma transformation were observed. The 2-year overall survival rate was（91.7±8.0）％, which did not significantly differ from that of the iHES group（96.2±2.6）％（*P*＝0.746）.

**Conclusion:**

Patients with L-HES generally have a favorable prognosis and are often characterized by skin involvement and significantly elevated serum IgE levels at diagnosis. They typically respond well to glucocorticoid therapy, although relapse is common during dose tapering. Interferon-alpha may serve as an effective second-line therapeutic option.

淋巴细胞变异型高嗜酸性粒细胞综合征（L-HES）在临床上较为罕见，是由异常免疫表型T淋巴细胞分泌嗜酸性粒细胞生长因子而引起的一种继发性HES[1]。1994年首次由Cogan等[2]报道，此后仅有个案或是小系列的病例报道[3]–[5]。我们总结了16例伴CD3^-^CD4^+^异常表型T细胞的L-HES患者的临床和实验室数据，以加深临床医师对该病的认识。

## 病例与方法

1. 病例：研究纳入2019年7月至2024年10月期间在中国医学科学院血液病医院骨髓增生异常综合征和骨髓增殖性肿瘤诊疗中心连续诊断的16例L-HES患者（研究组），65例同时期确诊的特发性HES（iHES）患者（对照组）。所有患者诊断参照2016年WHO造血与淋巴组织肿瘤分类标准[6]和文献[7]。HES的诊断标准需要满足外周血嗜酸性粒细胞绝对计数（AEoC）≥1.5×10^9^/L，并持续6个月以上（有器官受累需要及早治疗的患者除外），同时伴有嗜酸性粒细胞增多症导致的器官受累及功能异常。L-HES需要在满足HES标准的前提下，除外其他WHO定义的髓系或淋系肿瘤伴嗜酸性粒细胞增多症，同时通过多参数流式细胞术（MPFC）证实存在CD3^-^CD4^+^或CD3^+^CD4^-^CD8^-^或CD4^+^CD7^-^的异常表型T淋巴细胞群。iHES需要在满足HES标准的前提下，除外其他继发原因和WHO定义的髓系或淋系肿瘤伴嗜酸性粒细胞增多症。

2. 基线观察指标：初诊患者进行基线评估，包括完整的病史及体格检查，实验室检查包括：①血细胞计数及分类，心、肝、肾功能，便常规（盐水漂浮法检测寄生虫卵）；②血清维生素B_12_、血清免疫球蛋白定量、抗ENA抗体；③外周血流式细胞术：淋巴细胞免疫分型、细胞因子水平测定；④骨髓：涂片分类，骨髓病理活检和免疫组织化学染色，染色体核型分析，PDGFRA、PDGFRB、FGFR1和JAK2基因重排染色体荧光原位杂交检测，FIP1L1::PDGFRA融合基因检测，T细胞受体（TCR）γ/β基因重排检测，血液肿瘤相关基因靶向二代测序（NGS）分析，2022年开始增加全转录组RNA测序项目。此外需完善心电图和超声心动图、胸片、腹部超声检查，有必要者进行淋巴结、皮肤或肠镜活检。肝脏及脾脏肿大以肋缘下触及为阳性，血清免疫球蛋白（Ig）E正常水平<165.3 U/ml，血清维生素B_12_正常水平133～675 pmol/L，血清白细胞介素（IL）-5正常水平<3.1 ng/L。以上所有实验室检查均为本院病理中心自建项目。

3. 治疗方案：L-HES患者的一线治疗药物为糖皮质激素（曲安西龙、甲泼尼龙、泼尼松），初始剂量由临床医师根据患者的AEoC和临床症状来决定，通常起始剂量不超过泼尼松1.0 mg·kg^−1^·d^−1^。经糖皮质激素治疗无效或起效后泼尼松维持剂量≥10 mg/d的患者考虑参加临床试验或者联合二线用药，常用药物为α干扰素，常用剂量为300万单位隔日皮下注射，经一、二线药物治疗无效的患者考虑换用三线药物治疗，包括羟基脲（0.5～3 g/d）、伊马替尼（200～400 mg/d），以及环磷酰胺、长春新碱、依托泊苷等细胞毒药物。二、三线联合用药起效后逐渐将糖皮质激素减量至停用。iHES患者可接受羟基脲作为二线治疗药物，其他治疗原则同L-HES。

4. 疗效评价：参照文献[3]标准判定疗效。完全缓解（CR）：经过1个月治疗后AEoC降至正常（<0.5×10^9^/L），临床症状消失；部分缓解（PR）：经过1个月治疗后AEoC较基线值下降>50％但未达到正常水平，临床症状改善；未缓解（NR）：经过1个月治疗后AEoC稳定于基线值或升高。治疗总体有效率为CR率与PR率之和。

5. 随访：随访截止时间为2024年11月20日，诊断后L-HES组患者的中位随访时间为16（*IQR* 8～28）个月，iHES组为18（*IQR* 8～33.5）个月。随访资料来源于住院和门诊病历及电话随访记录。总生存（OS）时间定义为自诊断日期到死亡或末次随访日期。

6. 统计学处理：基线数值为连续变量的以中位数（*IQR*）描述，为分类变量的以频数（百分比）描述。对于连续变量（数据不符合正态分布），通过Mann-Whitney *U*检验进行患者亚组之间的比较。对于分类变量，通过*χ*^2^检验或Fisher确切概率法进行比较。双侧*P*<0.05被认为差异具有统计学意义。使用SPSS 22.0软件包进行统计分析。

## 结果

1. 人口学和临床特征：16例L-HES患者中，男9例，女7例，就诊时中位年龄为50.0（*IQR* 39.3～59.5）岁，同iHES组患者相比差异无统计学意义（表1）。L-HES患者就诊时最常见的受累器官分别为皮肤（75.0％）、胃肠道（25.0％）、呼吸道（18.8％）、淋巴结（18.8％）、心脏（12.5％）和脾脏（6.3％）。4例（25.0％）患者就诊时同时伴有两种器官损害，12例（75.0％）患者以单器官受累就诊。iHES患者就诊时最常见的受累器官分别为皮肤（41.5％）、胃肠道（35.4％）、神经肌肉（16.9％）、呼吸道（13.8％）、淋巴结（6.2％）和脾脏（3.1％）。两组除皮肤受累外（*P*＝0.016），其他受累器官差异无统计学意义（表1）。iHES组有9例患者以动静脉栓塞为主要临床表现就诊，但L-HES组未发现血栓栓塞的患者。乏力、发热、盗汗和体重减轻等全身症状在两组患者均少见。

**表1:** 淋巴细胞变异型高嗜酸性粒细胞综合征（L-HES）和特发性高嗜酸性粒细胞综合征（iHES）患者临床特征比较

临床特征	L-HES组 （16例）	iHES组 （65例）	统计量	*P*值
年龄［岁，*M*（*IQR*）］	50.0（39.3～59.5）	44.0（31.0～54.5）	*z*＝−1.412	0.158
性别（例，男/女）	9/7	36/29	*χ*^2^＝0.004	0.950
临床症状［例（％）］	
皮肤	12（75.0）	27（41.5）	*χ*^2^＝5.758	0.016
胃肠道	4（25.0）	23（35.4）	*χ*^2^＝0.623	0.430
呼吸道	3（18.8）	9（13.8）	*χ*^2^＝1.231	0.919
浅表淋巴结肿大	3（18.8）	4（6.2）	*χ*^2^＝0.004	0.267
心脏	2（12.5）	4（6.2）	*χ*^2^＝0.745	0.338
脾脏肿大	1（6.3）	2（3.1）	*χ*^2^＝0.318	0.488
神经肌肉	0（0）	11（16.9）	*χ*^2^＝1.857	0.173
血栓	0（0）	9（13.8）	*χ*^2^＝1.288	0.257
全身症状	0（0）	1（1.5）	*χ*^2^＝0.000	1.000
WBC［×10^9^/L，*M*（*IQR*）］	11.7（7.5～18.3）	10.9（8.1～14.2）	*z*＝−0.343	0.732
HGB［g/L，*M*（*IQR*）］	134（122～146）	142（130～154）	*z*＝−1.270	0.204
PLT［×10^9^/L，*M*（*IQR*）］	244.5（220.3～284.5）	243.5（190.3～295）	*z*＝−0.126	0.899
ALynC［×10^9^/L，*M*（*IQR*）］	1.9（1.3～3.9）	2.3（1.7～3.1）	*z*＝−0.255	0.799
AEoC［×10^9^/L，*M*（*IQR*）］	4.1（1.4～9.6）	2.4（1.4～4.7）	*z*＝−1.196	0.232
嗜酸性粒细胞百分比［％，*M*（*IQR*）］	44.7（17.6～59.4）	23.9（16.4～42.9）	*z*＝−1.708	0.088
血清维生素B_12_［pmol/L，*M*（*IQR*）］	237.5（149.8～403.8）	262.0（179.0～340.5）	*z*＝−0.213	0.831
维生素B_12_水平升高［例（％）］	0（0）	2（3.1）	*χ*^2^＝0.000	1.000
血清IgE［IU/ml，*M*（*IQR*）］	1 885（304～4 502）	122（38～397）	*z*＝−3.840	<0.001
IgE水平升高［例（％）］	13（81.3）	23（35.4）	*χ*^2^＝10.939	0.001
血清IL-5［ng/L，*M*（*IQR*）］	5.3（4.1～8.0）	4.1（2.3～7.1）	*z*＝−1.571	0.116
IL-5水平升高［例（％）］	13（81.3）	28（63.6）^a^	*χ*^2^＝1.682	0.195
TCR重排［例（％）］	12（75.0）	2（3.1）	*χ*^2^＝46.457	<0.001
随访时间［月，*M*（*IQR*）］	16（8～28）	18（8～33.5）	*z*＝−0.465	0.642
死亡［例（％）］	1（6.3）	3（4.6）	*χ*^2^＝0.000	1.000

**注** ALynC：外周血淋巴细胞绝对计数; AEoC：外周血嗜酸性粒细胞绝对计数；IL-5：白细胞介素-5；TCR：T细胞受体。^a^44例iHES患者检测了血清IL-5水平

2. 实验室特征：L-HES组患者就诊时的中位WBC为11.7（7.5～18.3）×10^9^/L，中位AEoC为4.1（1.4～9.6）×10^9^/L，中位嗜酸性粒细胞百分比为44.7％（17.6％～59.4％），两组患者的全血细胞计数各项参数差异无统计学意义（表1）。通过MPFC检测，L-HES患者外周血中CD3^-^CD4^+^T细胞占淋巴细胞中位百分比为4.08％（1.64％～32.78％），中位绝对计数为0.10（0.05～0.55）×10^9^/L。所有iHES患者均未检测到CD3^-^CD4^+^T细胞群。

两组患者的血清维生素B_12_和IL-5水平差异无统计学意义。L-HES组患者血清IgE水平显著高于iHES组（*P*<0.001）。75.0％的L-HES患者检测到TCRγ或TCRβ至少一种克隆性重排。虽然2例（3.1％）iHES患者也检测到克隆性TCR重排，但MPFC未发现表型异常的T淋巴细胞（表1）。

5例L-HES患者通过NGS检测到致病性基因突变，其中1例患者为SBDS c.T258+2C，变异等位基因频率（VAF）为43.92％，经毛囊细胞验证为胚系来源。另外4例（30.8％）患者检测到7种突变基因，VAF均<10％。突变基因依次为DNMT3A 2例，GNB1、KRAS、MPL、RELN和TET2各1例。此外，1例患者通过RNA测序检测到ITK::SYK融合基因（表2）。13例（20％）iHES患者检测到了10种突变基因，依次为DNMT3A 7例，TET2 2例，ASXL1、ARID1A、BCOR、BRAF、JAK2、IDH2、KMT2D、ZRSF2各1例。除1例BCOR突变的VAF为29.2％，其余突变的VAF均<10％。两组患者的克隆性造血检出率差异无统计学意义（*P*＝0.464）。

**表2:** 5例二代测序异常的淋巴细胞变异型高嗜酸性粒细胞综合征患者生物学特征和结局

例号	性别	年龄（岁）	主要就诊原因	CD3^-^CD4^+^占淋巴细胞百分比（绝对计数，×10^9^/L）	TCR重排	IgE（IU/ml）	IL-5（ng/L）	基因突变（VAF）	治疗和随访
1	女	52	周身皮疹伴瘙痒13年，间断性腹泻、咳嗽8年	1.92％（0.05）	TCRβ（−） TCRγ（−）	1 530	2.01	SBDS-c.T258+2C（43.92％）	泼尼松30 mg/d，获得CR，减量至10 mg/d，嗜酸性粒细胞再次增多并出现皮肤症状。目前入组临床试验，尚未揭盲
2	男	75	间断皮疹伴瘙痒12年，乏力伴双下肢水肿1年	44.38％（0.69）	TCRβ（+） TCRγ（−）	2 440	5.10	KRAS-p.G13C（2.8％）；DNMT3A-p.Q656Hfs*7（2.3％）	根据临床症状间断口服泼尼松5～20 mg/d，5 mg/d可维持CR，停药后复发。患者二、三尖瓣中重度反流，就诊后8个月因心功能不全死亡
3	女	60	皮疹伴瘙痒16年，发现嗜酸性粒细胞升高10余年，皮肤活检提示嗜酸性粒细胞浸润	18.91％（0.27）	TCRβ（+） TCRγ（+）	5 049	6.48	MPL-p.R592*（6.4％）；GNB1-p.K57E（1.2％）；DNMT3A-p.F909I（2.2％）	甲泼尼龙16 mg/d，获得CR，减量至2 mg/d可维持缓解，停药后嗜酸性粒细胞再次增多伴皮肤瘙痒，自行服用依巴斯汀治疗
4	女	49	间断皮疹伴瘙痒15个月，皮肤活检提示嗜酸性粒细胞浸润，应用雷公藤多苷片、他克莫司和沙利度胺治疗症状无改善	37.40％（0.45）	TCRβ（+） TCRγ（+）	185	4.96	RELN-p.T759I（2.2％）	曲安西龙24 mg/d获得CR，目前减量至4 mg隔日，维持嗜酸性粒细胞正常，仍有间断皮肤瘙痒
5	女	62	全身皮肤瘙痒，反复出现紫红色皮疹，气促、双下肢水肿4个月，诊断心功能不全，限制性心肌病	4.55％（0.05）	TCRβ（+） TCRγ（+）	2 700	8.28	RNA测序检测到ITK::SYK融合基因	完善PET-CT和颈部淋巴结活检，不支持淋巴瘤诊断，曲安西龙28 mg/d，获得CR，目前曲安西龙减量至4 mg/d维持1年，嗜酸性粒细胞维持（0.5～1）×10^9^/L，偶有皮疹伴瘙痒

**注** TCR：T细胞受体；IL-5：白细胞介素-5；VAF：变异等位基因频率；CR：完全缓解

3. 治疗和随访：14例患者诊断后接受糖皮质激素作为一线治疗。中位初始剂量相当于泼尼松30（20～42.5）mg/d，11例患者获得CR，2例患者获得PR，糖皮质激素作为L-HES一线治疗的总体有效率为92.9％。2例获得PR的患者中，例5因皮肤症状未缓解，先后接受包括联合化疗在内的多线治疗，目前应用度普利尤单抗治疗，皮肤症状缓解。例14联合干扰素α2b治疗后获得CR。1例NR患者因临床症状改善，仍继续接受糖皮质激素治疗。

糖皮质激素减量过程中，11例患者再次出现嗜酸性粒细胞计数增多或临床症状加重。仅3例患者在疾病复发时接受了二线治疗，其中1例入组临床试验，结果尚未揭盲，另外2例患者在联合α干扰素治疗后均再次获得CR。至随访终点，8例患者继续接受糖皮质激素维持治疗，中位维持剂量相当于泼尼松5.0（4.4～11.3）mg/d。

L-HES组患者就诊前的中位病程为30（12～117）个月，就诊后中位随访16（*IQR* 8～28）个月。1例患者失访，1例患者就诊后8个月死于心脏功能不全。iHES组患者就诊前的中位病程为12（3～48）个月，就诊后中位随访18（*IQR* 8～33.5）个月。8例患者失访，3例患者死亡，均死于血栓并发症，包括1例患者发生脑梗死，1例发生心肌梗死，1例出现心室附壁血栓。L-HES组患者的预计2年OS率为（91.7±8.0）％，iHES组为（96.2±2.6）％，两组间差异无统计学意义（*P*＝0.746）（图1）。

**图1:**
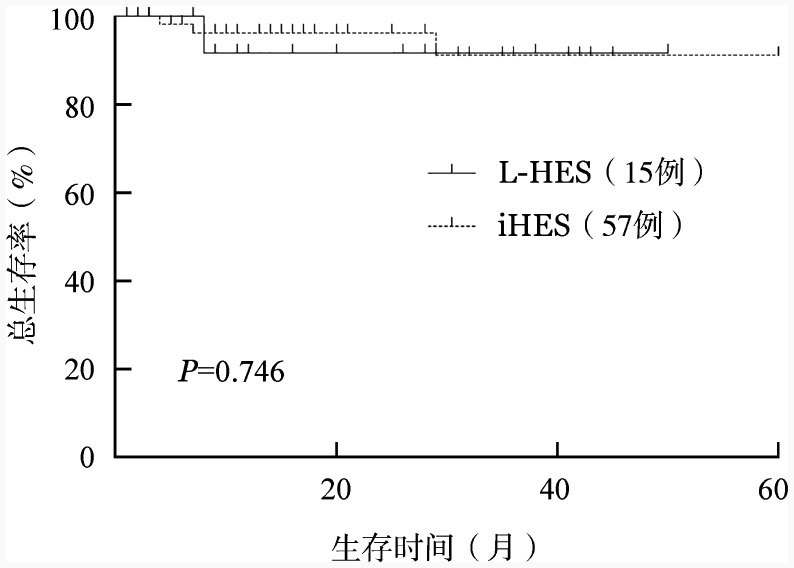
淋巴细胞变异型高嗜酸性粒细胞综合征（L-HES）和特发性高嗜酸性粒细胞综合征（iHES）患者的总生存比较

## 讨论

L-HES是1994年首次由Cogan等[2]报道，1例30岁男性HES患者，以广泛性皮肤瘙痒症状起病，外周血发现一群CD3^-^CD4^+^ T淋巴细胞，患者在接受干扰素治疗后嗜酸性粒细胞计数恢复正常，CD3^-^CD4^+^淋巴细胞比例下降。随后Simon等[8]通过对16例此类患者的研究明确建立了L-HES的概念，证明Th2细胞过度产生IL-5是导致患者血液和组织中嗜酸性粒细胞增多的主要原因，因此将L-HES归属于继发性HES的范畴。L-HES患者就诊时可以表现为多种器官受累，我们的研究结果显示皮肤症状是L-HES患者最常见的就诊原因，这同既往的文献报道[2]–[5]相一致。其他受累器官同iHES组差异无统计学意义。L-HES患者的皮肤表现差异性极大，可从轻微的短暂性皮疹到全身性红皮病不等。单个患者可能同时出现多种皮肤表现，包括弥漫性瘙痒、湿疹样病变、丘疹样荨麻疹、斑丘疹、红皮病和面部血管神经性水肿等[9]。除皮肤病变外，文献报道其他常见表现还包括周期性血管神经性水肿、浅表淋巴结肿大、气道高敏症状、合并自身免疫性疾病等，但除皮肤症状以外，其他常见临床表现在各项研究中并不一致[4]–[5],[10]。

L-HES目前仍缺乏统一的诊断标准，在满足HES诊断标准的前提下，应以检测到免疫表型异常的T淋巴细胞为主要依据，最常见的表型是CD3^-^CD4^+^，此外也有个案报道CD3^+^CD4^-^CD8^-^和CD4^+^CD7^-^的免疫表型[11]–[12]，我们报道的16例L-HES患者均为典型的CD3^-^CD4^+^免疫表型。我们的研究结果显示，多数患者就诊时异常淋巴细胞比例较低，这也要求流式细胞术分析人员提高警惕，以免漏诊。TCR基因重排检测是临床上最常用的T细胞克隆性分析方法，但并非所有L-HES患者均可检测到克隆性TCR重排[2]。我们的研究中，有25％的L-HES患者TCR重排阴性，主要集中在异常细胞群占淋巴细胞比例<2％的患者中，这提示阴性结果可能同检测方法灵敏度相关，此前Lefèvre等[4]的研究也有类似的发现。此外，有2例iHES患者检测到TCR基因克隆性重排，但由于TCR基因重排可以在一些老年人、自身免疫性疾病及病毒感染的患者中被检测到，也可以在各种HES亚型中被检测到，因此目前诊断L-HES的主要依据是异常淋巴细胞免疫表型，而非TCR基因重排[1]。理论上Th2细胞分泌过多的IL-5是导致嗜酸性粒细胞增多的主要原因，但我们的研究显示，3例L-HES患者的血清IL-5水平并未升高，而相比iHES组，L-HES组患者的IL-5中位水平虽然更高，但两组间差异并无统计学意义。血清细胞因子检测受多种因素影响，经典的方法是检测经体外24 h培养的单个核细胞上清液中的IL-5水平[8]，但该方法难以在临床广泛应用，因此目前并未将血清IL-5水平作为L-HES的必要诊断条件[1]。在各项实验室检查中，两组患者的血清IgE水平差异最显著，相比iHES组，L-HES患者有更高的血清IgE水平。虽然有4例患者检测到克隆性造血，但VAF值均在10％以下，其中3例接受糖皮质激素治疗的患者均获得了CR，因此我们推测这些克隆性造血同嗜酸性粒细胞增多症并无直接相关。

糖皮质激素是目前L-HES最常用的治疗药物，各项研究报道的反应率在85％～100％[13]。虽然一项回顾性分析结果显示L-HES是糖皮质激素疗效差的一项风险预测因素[14]，但该结果并未获得其他研究的支持[4]–[5]。我们的研究中，接近93％的患者对糖皮质激素治疗有反应，多数患者可以在较低的初始剂量下（相当于泼尼松<0.5 mg·kg^−1^·d^−1^）获得快速的血液学和临床症状缓解。但多数患者减量过程中会出现复发，由于病程长和反复发作的特点，患者常自行将剂量维持在一种可耐受症状的水平，并不追求嗜酸性粒细胞计数完全恢复正常。干扰素是目前最常用的二线治疗药物，通常选择性应用于糖皮质激素治疗无效或者需要较大维持剂量的患者[15]。一项研究报道8例L-HES患者在接受聚乙二醇干扰素治疗后均获得临床反应，其中6例患者在1个月内减停了糖皮质激素[15]。在我们的研究中，3例患者接受了干扰素治疗，其中2例获得CR。其他文献报道的有效治疗药物还包括美泊利珠单抗、环孢素和麦考酚酸酯等[16]–[18]，可作为二、三线治疗选择。度普利尤单抗是一种IL-4受体α亚基拮抗剂，目前国内获批用于中重度特应性皮炎，由于IL-4也是一种嗜酸性粒细胞生长因子，因此理论上对L-HES引起的皮肤损害有效，我们的1例患者在接受多线药物治疗无效后，应用度普利尤获得临床缓解。目前已有一些应用度普利尤单抗治疗HES的个案报道[19]，将来有望成为糖皮质激素的替代药物。

L-HES诊断时与T细胞淋巴瘤的鉴别以及长期淋巴瘤的转化风险一直是临床关心的重点，法国嗜酸性粒细胞工作组的一项报道显示有10％（5/49）左右的患者在中位随访9（4～15）年的时间里转化为外周T细胞淋巴瘤，以血管免疫母性T细胞淋巴瘤最为常见[20]。我们报道的16例患者虽然没有发现淋巴瘤转化的病例，但有2例患者在外院检查时曾怀疑为淋巴瘤，最终经过多部位活检和PET-CT检测排除了淋巴瘤的诊断。1例患者检测到ITK::SYK融合基因，这是一种常见于外周T细胞淋巴瘤的基因重排，虽然通过全身PET-CT和淋巴结活检未能确定淋巴瘤的诊断，但我们仍对该例患者保持密切随访。

综上，L-HES患者就诊时容易表现为皮肤受累和血清IgE显著升高，患者通常对糖皮质激素治疗较敏感，但减量过程中容易出现复发，干扰素是一种有效的二线治疗药物。患者总体预后良好，由于本研究是基于单中心回顾性研究，病例数相对较少，有待下一步全国多中心、前瞻性研究来加以进一步确证。
